# Analysis of topical conjunctival microbiotic cultures in patients treated with intravitreal injections using antibiotic prophylaxis with 0.3% ofloxacin eye drops

**DOI:** 10.1186/s40942-024-00604-x

**Published:** 2024-12-20

**Authors:** Luca Bongiovanni de Miranda Gonçalves, Maria Leticia Lasca Sales Campos, Guilherme Feltrin Barros, Glaucia Luciano da Veiga, Juliana Antoniali Silva, Fernando Luiz Affonso Fonseca, Thaís Moura Gascón, Samantha Sanches de Carvalho, Andrea Karla Ribeiro de Carvalho, Greicy Ellen Pinheiro Fernandes, Vagner Loduca Lima, Tiago Mirco Lima

**Affiliations:** 1 Centro Universitário Faculdade de Medicina Do ABC/FMABC, Santo André, Brazil; 2 Universidade Federal de São Paulo/UNIFESP, São Paulo, Brazil; 3 Universidade de Campinas/UNICAMP, Campinas, Brazil

**Keywords:** Endophthalmitis, Conjunctival microbiota, Intravitreal injection

## Abstract

**Background:**

Intravitreal injections, a relatively recent treatment in ophthalmology, is being adopted rapidly worldwide and becoming one of the most common therapies in the field. Numerous complications are associated with this treatment, ranging from minor inflammatory ailments to endophthalmitis. We analyzed the conjunctival flora of patients treated with intravitreal injections and topical antibiotics.

**Methods:**

The study was a longitudinal prospective analysis of cultures and antibiograms collected from patients who underwent intravitreal injections and topical antibiotics afterwards at the retina clinic in ABC’s Medical University.

**Results:**

A total of 148 swabs obtained from 98 patients were cultured and underwent antibiotic sensitivity testing. All bacteria were sensitive to vancomycin, and with the exception of *Escherichia coli* samples, they were also sensitive to a third-generation cephalosporin (ceftriaxone—same class and generation as ceftazidime), both of which are important antibiotics for the treatment of endophthalmitis. The bacteria species were specifically coagulase-negative Staphylococcus sp. 92% of which was penicillin-resistant and 56.9% was resistant to ciprofloxacin, a second-generation fluoroquinolone. The culture results were similar to that described in the literature and showed the same higher prevalence of coagulase-negative *Staphylococcus sp.* and *S. epidermidis.* Regarding the antibiotic resistance profiles, vancomycin, a third-generation cephalosporin, and penicillin showed almost identical results to those reported previously. Regarding fluoroquinolones, the incidence of resistant coagulase-negative *Staphylococcus sp.* was lower than the findings worldwide, but the resistance rates found were: *S. aureus* (26.7%), *S. epidermidis* (61.3%), and *Staphylococcus sp*. (coagulase negative, 56.9%).

**Conclusions:**

The current results showed that the typical conjunctival bacteria had higher resistance to fluoroquinolones (although they were not tested specifically to ofloxacin), suggesting a possible selection of resistant bacteria that should not be taken for granted in clinic. However, the same bacteria did not exhibit cross-resistance in the analysis of vancomycin and third-generation cephalosporins. This real-world, longitudinal, prospective study on conjunctival flora analyzed bacterial resistance profiles and contemporary antibiotic use, offering deeper insights into this subject.

## Background

The use of intravitreal injections is rapidly expanding and becoming widespread across ophthalmology centers worldwide. The first injection of air into the vitreous cavity was reported by Ohm in 1911 in a case of rhegmatogenous retinal detachment [1]. The use of antibiotic injections into the vitreous has appeared in the literature since the 1940s [2]. From 1998 onward, the United State Food and Drug Administration authorized the injection of fomivirsen (Vitravene, Ionis Pharmaceuticals, Carlsbad, CA, USA; 6 years after that, pegaptanib (Macugen, Bausch & Lomb, Bridgewater, NJ, USA), in 2006 ranibizumab (Lucentis, Genentech Inc., South San Francisco, CA, USA), and the off-label use of bevacizumab (Avastin, Genentech Inc.) since 2005 [3, 4]. New formulations have been developed and the literature increasingly reflects the effectiveness of, for example, ranibizumab and triamcinolone for treating macular edema after central retinal vein occlusion [5, 6].^.^

With the dissemination of intravitreal injections worldwide and the acceptance by the global ophthalmic community, discussions began about the complications of this new therapeutic tool, among which are rhegmatogenous retinal detachment with an incidence of up to 0.45% and acute intraocular inflammation and endophthalmitis, which can reach an incidence of 1% [7–12]. Unlike cataract surgery, about which randomized clinical studies have proven the benefits of topical postoperative antibiotic prophylaxis, such as that developed by the European Society of Refractive and Cataract Surgery, no studies have demonstrated the beneficial effect of their prophylactic use after injections [13]. A 2016 meta-analysis contraindicated the use of antibiotics; of 174,159 intravitreal injections, the incidence rates of endophthalmitis with/without post-injection antibiotic prophylaxis, respectively, were 0.052% and 0.048% [14]. Another systematic review reported a threefold higher incidence of endophthalmitis in those in whom topical antibiotics were used after intravitreal injections compared to the control group [15].

With the adoption by various ophthalmologic services of intravitreal injections worldwide, there have been a growing number of studies, routines, and clinical manuals on infectious prophylaxis. The literature describes the use of some pre-injection topical antiseptic agents, such as 5% povidone-iodine solution (PVPI) and post-injection topical antibiotics. In 2021, Celebi and Celebi reported that the coagulase-negative *Staphylococcus* strains associated with administration of topical quinolones 4 times daily for 7 days after intravitreal injections were significantly resistant to the antibiotics [16]. Furthermore, there are a number of strains resistant to a wide range of topical antibiotics used prophylactically [17]. In 1997, Kunisada et al. reported that bacteria were resistant to chlorhexidine studied in vitro [18]. However, PVPI, not associated with the use of topical antibiotics, did not promote the selection of conjunctival microbiota with changes in the resistance pattern [19]. Furthermore, in 2009, Moss et al. found that PVPI alone resulted in a dramatic reduction in the number of positive cultures and confirmed the lack of benefit from the use of topical antibiotics [20]. Regarding the use of antibiotics before intravitreal injections, a 2017 study reported no clinical impact on the prevention of endophthalmitis or the reduction of the conjunctival microbiota, with no additional benefits from the use of PVPI [21, 22]. PVPI also has demonstrated has bactericidal effects equivalent to the use of topical antibiotics for 3 days [23].

The rampant use of antibiotics in medicine has created the emergence of numerous multidrug resistant infections. The selection of resistant microbiota at an alarming rate worldwide has become an important target for studies that the promote awareness of the medical community to the flagrant use of antibiotics [24, 25]. In cultures of endophthalmitis, resistant strains are increasingly found and they are demonstrating more virulence [26]. It is essential to better understand how to reduce this growth in resistant and more virulent bacteria, so that the treatment of endophthalmitis does not become even more challenging.

The objective of the current study was to analyze the conjunctival microbiota of patients at the retina outpatient clinic of FMABC ophthalmology undergoing intravitreal injections who had been using 0.3% ofloxacin eye drops for infectious prophylaxis.

## Methods

This prospective longitudinal study was performed from May 15, 2023, to August 14, 2023, with patients from the outpatient clinic of the retina sector of the medical residency service of the ophthalmology discipline of the medical school of the ABC/FMABC (Faculdade de Medicina do ABC) health university center, in Santo André, a metropolitan region of São Paulo, Brazil. The patients provided written consent in accordance with the Research Ethics Committee of the National Health Council (Annex I), and the Ethics and Research Committee of Centro Universitário de Saúde ABC/FMABC approved the study protocol (CAAE: 68459623.6.0000.0082).

All participants received the Free and Informed Consent Form to confirm that they agreed to participate in the study. All measures regarding the freedom of participation of the individual to be studied and their integrity throughout the study were guaranteed. All data that could identify them were masked. During the study, there was no protocol or situation that could harm the privacy, secrecy, or confidentiality of the patient and their personal information.

Patients aged over 18 years being treated at the Retina outpatient clinic who had indications for intravitreal injections were recruited. All patients were prescribed ofloxacin 0.3% eye drops every 6 h for 7 days after their injections. An equal number of males and females were recruited in the study. Other factors such as gender, color/race or ethnicity, classes or social groups were not factors delimiting the studied population.

The exclusion criteria included patients who had not used ofloxacin 0.3% eye drops; were already using antibiotic eye drops; had undergone another recent ophthalmologic procedure; were using oral antibiotics; were immunocompromised; presented phlogistic at the time of collection; missed or could not complete any of the applications; did not sign the informed consent form, or withdrew from study participation.

Swabs were used to collect the samples 30 days after the intravitreal injections, for all participants. Anesthetic eye drops (tetracaine hydrochloride 10 mg/ml + phenylephrine 1 mg/ml, 10 ml) were instilled prior to the swab. Materials were collected from the conjunctiva of the lower fornix before asepsis or antisepsis. Collection took place using gloves to handle the swab and sterile cotton swabs that were gently rubbed on the tarsal and inferior bulbar conjunctiva, with care taken to not contaminate the material before storage in its appropriate and sealed reservoir. The culture kit included Stewart's medium. The culture medium was taken to the laboratory at the ABC Medical School. The college laboratory personnel performed the culturing of the swabs on Hagar blood and chocolate. The antibiograms were performed after the culture was positive with the focus on the following antibiotics: azithromycin, clarithromycin, erythromycin, penicillin, ciprofloxacin, oxacillin, and clindamycin. The calculation of the minimum inhibitory concentration (MIC) was performed from the double disk diffusion test on the Phoenix machine (Becton Dickinson, Franklin Lakes, NJ, USA). The samples were classified as sensitive, intermediate, or resistant according to the MIC and the diameter of the halo observed. The references used were the values found in the tables of the Brazilian Committee on Antimicrobial Susceptibility Testing and the European Committee on Antimicrobial Susceptibility Testing.

Intravitreal injections were performed in the surgical center of the same outpatient clinic. The doctors who administered the injections were ophthalmology residents and retina fellows from the ophthalmology service of the ABC Medical School in accordance with the institution’s protocols, i.e., in a sterile surgical center with the patient in a horizontal supine position, and with asepsis and antisepsis using 5% PVPI. Surgeons wore sterile gloves, cap, mask and a sterile surgical gown. A sterile, disposable fenestrated drape was placed, followed by a sterile single-piece wired blepharostat. A 1-mL syringe and a sterile 30-gauge hypodermic needle were used to inject the substance 3.5 or 4.0 mm from the limbus into the superior or inferior temporal region. After application, the visual acuity was checked by the clinician moving his or her hand in front of the patient and then topical PVPI 5% was instilled and the blepharostat and fenestrated field were removed. Along with the instructions, all patients received a prescription for 0.3% ofloxacin eye drops 4 times daily for 7 days.

In this study, a descriptive statistical analysis was performed to examine the sample characteristics. Categorical variables were analyzed regarding frequency; for continuous variables, the mean and standard deviation were calculated. The approach was exclusively descriptive, without application of statistical tests for comparisons between groups.

## Results

A total of 98 participants (50% men) were evaluated. Table 1 shows the gender, eye laterality, and number of applications by eye. For sex and laterality, the percentage was calculated in relation to the total number of participants. The male prevalence was 50%; both eyes of 50% of patients were included; and three or more applications were administered to both eyes in 80%. Another 16 patients who met the inclusion criteria refused participation and were excluded. Half of the patients had samples collected from both eyes, 23 (23.5%) only from the right eye, and 26 (26.5%) only from the left eye.

**Table 1:** Patient data

	No	%
No. participants	98	
Sex, n (%)		
Female	49	50.0
Male	49	50.0
Ocular laterality, no. (%)		
Both eyes	49	50.0
Right eye	23	23.5
Left eye	26	26.5

Right eye		
< 3 applications	12	16.4
3 or more applications	61	83.6
Left eye		
< 3 applications	8	10.7
3 or more applications	67	89.3

No. applications by eye, no. (%)

Regarding the number of applications per laterality, the percentage was calculated in relation to the total number of participants with applications for each laterality

A total of 148 samples were collected from these patients. Sixteen samples (10.81%) had negative cultures. In addition to the negative samples, antibiograms were not performed on another 20 cultures (13.51%), totaling 36 samples (24.32%) without antibiograms. A total of 128 (86.48%) samples were collected from patients with at least three previous intravitreal applications and 20 (13.51%) had fewer than three. 50% of the samples were from patients who had one swab collected from each eye at different times, as the intravitreal injections were never administered simultaneously. The culture results grew 20 different microorganisms, the most frequent of which were *Staphylococcus sp*. (coagulase-negative), *S. epidermidis*, *S. aureus*, *Corynebacterium sp.,* and *S. haemolyticus* (Tables 2, 3). *Staphylococcus sp.* (coagulase-negative) and *S. epidermidis* comprised 93.1% and 95.5%, respectively, of the samples.

**Table 2:** Percentage of cultures in the eye

	% composition in the eye (%)	
	No	Sent to antibiotic sensitivity testing (SD - Mean)
*Staphylococcus sp. (coagulase negative)*	54	93.1 (19.6)
*S. epidermidis*	33	95.5 (13.3)
*S. aureus*	15	89.3 (28.1)
*Corynebacterium sp.*	12	69.2 (33.2)
*S. haemolyticus*	12	86.7 (31.1)
*Streptococcus sp. grupo viridans*	6	76.7 (18.6)
*Acinetobacter baumannii-calcoacetius complex*	3	100.0 (0.0)
*Klebsiella oxytoca*	3	93.3 (11.5)
*Enterococcus faecalis*	2	85.0 (21.2)
*Escherichia coli*	2	100.0 (0.0)
*K. pneumoniae*	2	100.0 (0.0)
*Acinetobacter lwoffii*	1	30.0 (–)
*Morganella morganii*	1	100.0 (–)
*Serratia marcescens*	1	100.0 (–)
*S. capitis*	1	100.0 (–)
*S. hominis*	1	100.0 (–)
*S. saprophyticus*	1	100.0 (–)
*Streptococcus sp grou D (no enterococcus)*	1	20.0 (–)
*Streptococcus sp. group D*	1	100.0 (–)
*Viridans Streptococcus*	1	50.0 (–)

**Table 3:** Analysis of the composition of microorganisms by eye

	n
*Staphylococcus sp. (coagulase negative)*	44
*S. epidermidis*	28
*S. aureus*	13
*S. haemolyticus*	9
*Corynebacterium sp.*	4
*Acinetobacter baumannii-calcoacetius complex*	3
*Corynebacterium sp., Staphylococcus sp. (coagulase negative)*	3
*Corynebacterium sp., S. epidermidis*	2
*Corynebacterium sp., S. haemolyticus*	2
*Escherichia coli*	2
*Klebsiella oxytoca*	2
*K. pneumoniae*	2
*S. aureus, viridans group Streptococcus sp.*	2
*S. sp. (coagulase negativo), viridans group Streptococcus sp.*	2
*Acinetobacter lwoffii, Staphylococcus epidermidis*	1
*Corynebacterium sp., Klebsiella oxytoca*	1
*Enterococcus faecalis, Staphylococcus sp. (coagulase negativo)*	1
*E. faecalis, Staphylococcus sp. (coagulase negativo), Staphylococcus sp. (coagulase negative), viridans group Streptococcus sp.*	1
*Morganella morganii*	1
*Serratia marcescens*	1
*S. capitis, S. haemolyticus, Streptococcus sp. group D*	1
*S. epidermidis, Staphylococcus sp. (coagulase negative)*	1
*S. epidermidis, viridans group Streptococcus sp.*	1
*S. hominis*	1
*S. saprophyticus*	1
*Staphylococcus sp. (coagulase negative), Streptococcus sp. group D (no enterococcus)*	1
*Viridans group Streptococcus sp.*	1

Table 3 shows that the compositions of the type of culture existing in the same eye presented in decreasing order of frequency, with *Staphylococcus sp.* (coagulase-negative) the most common.

Tables 4, 5, 6, 7, 8, 9, 10, 11, 12, 13, 14, 15, 16, 17, 18, 19, 20, 21, 22, 23, 24, 25, 26, 27, 28, 29 show the antibiotic sensitivity by microorganism described as antibiotic sensitive, intermediate, or resistant. The percentage was calculated based on the total number of participants who underwent the antibiogram for a given type of culture and had results available.

**Table 4:** Microorganism sensitivity to amikacin

	Sensitive (%)	Intermediate (%)	Resistant (%)
*Acinetobacter baumannii-calcoacetius complex*	3 (100.0)		
*A. lwoffii*	1 (100.0)		
*Escherichia col*	2 (100.0)		
*Klebsiella oxytoca*	3 (100.0)		
*K. pneumoniae*	2 (100.0)		
*Morganella morganii*	1 (100.0)		
*Serratia marcescens*	1 (100.0)		

Frequency calculated in relation to the participants who performed the antibiogram

**Table 5:** Microorganism sensitivity to amoxicillin

	Sensitive (%)	Intermediate (%)	Resistant (%)
*Enterococcus faecalis*	2 (100.0)	0 (0.0)	0 (0.0)
*Escherichia coli*	1 (50.0)	0 (0.0)	1 (50.0)
*Klebsiella oxytoca*	3 (100.0)	0 (0.0)	0 (0.0)
*K. pneumoniae*	2 (100.0)	0 (0.0)	0 (0.0)
*Morganella morganii*	0 (0.0)	0 (0.0)	1 (100.0)
*Serratia marcescens*	0 (0.0)	0 (0.0)	1 (100.0)
*Viridans group Streptococcus sp.*	3 (100.0)	0 (0.0)	0 (0.0)

Frequency calculated in relation to the participants who performed the antibiogram

**Table 6:** Microorganism sensitivity to ampicillin

	Sensitive (%)	Intermediate (%)	Resistant (%)
*Enterococcus faecalis*	2 (100.0)	0 (0.0)	0 (0.0)
*Klebsiella oxytoca*	0 (0.0)	0 (0.0)	3 (100.0)
*K. pneumoniae*	0 (0.0)	0 (0.0)	2 (100.0)
*Morganella morganii*	0 (0.0)	0 (0.0)	1 (100.0)
*Serratia marcescens*	0 (0.0)	0 (0.0)	1 (100.0)
*Viridans group Streptococcus sp.*	3 (100.0)	0 (0.0)	0 (0.0)

Frequency calculated in relation to the participants who performed the antibiogram

**Table 7:** Microorganism sensitivity to azithromycin

	Sensitive (%)	Intermediate (%)	Resistant (%)
*Corynebacterium sp.*	1 (100.0)	0 (0.0)	0 (0.0)
*Staphylococcus aureus*	1 (6.7)	0 (0.0)	14 (93.3)
*S. capitis*	0 (0.0)	0 (0.0)	1 (100.0)
*S. epidermidis*	9 (29.0)	0 (0.0)	22 (71.0)
*S. haemolyticus*	5 (41.7)	0 (0.0)	7 (58.3)
*S. hominis*	0 (0.0)	0 (0.0)	1 (100.0)
*S. saprophyticus*	1 (100.0)	0 (0.0)	0 (0.0)
*Staphylococcus sp. (coagulase negative)*	20 (39.2)	0 (0.0)	31 (60.8)

Frequency calculated in relation to the participants who performed the antibiogram

**Table 8:** Microorganism sensitivity to cefepime

	Sensitive (%)	Intermediate (%)	Resistant (%)
*Escherichia coli*	1 (50.0)	0 (0.0)	1 (50.0)
*Klebsiella oxytoca*	3 (100.0)	0 (0.0)	0 (0.0)
*K. pneumoniae*	2 (100.0)	0 (0.0)	0 (0.0)
*Morganella morganii*	1 (100.0)	0 (0.0)	0 (0.0)
*Serratia marcescens*	1 (100.0)	0 (0.0)	0 (0.0)
*Viridans group Streptococcus sp.*	3 (100.0)	0 (0.0)	0 (0.0)

Frequency calculated in relation to the participants who performed the antibiogram

**Table 9:** Microorganism sensitivity to cefotaxime

	Sensitive (%)	Intermediate (%)	Resistant (%)
*Escherichia coli*	1 (50.0)	0 (0.0)	1 (50.0)
*Klebsiella oxytoca*	3 (100.0)	0 (0.0)	0 (0.0)
*K. pneumoniae*	2 (100.0)	0 (0.0)	0 (0.0)
*Morganella morganii*	1 (100.0)	0 (0.0)	0 (0.0)
*Serratia marcescens*	1 (100.0)	0 (0.0)	0 (0.0)
*Viridans group Streptococcus sp.*	3 (100.0)	0 (0.0)	0 (0.0)

Frequency calculated in relation to the participants who performed the antibiogram

**Table 10:** Microorganism sensitivity to ceftriaxone

	Sensitive (%)	Intermediate (%)	Resistant (%)
*Escherichia coli*	1 (50.0)	0 (0.0)	1 (50.0)
*Klebsiella oxytoca*	3 (100.0)	0 (0.0)	0 (0.0)
*K. pneumoniae*	2 (100.0)	0 (0.0)	0 (0.0)
*Morganella morganii*	1 (100.0)	0 (0.0)	0 (0.0)
*Serratia marcescens*	1 (100.0)	0 (0.0)	0 (0.0)
*Viridans group Streptococcus sp.*	3 (100.0)	0 (0.0)	0 (0.0)

Frequency calculated in relation to the participants who performed the antibiogram

**Table 11:** Microorganism sensitivity to cefuroxime

	Sensítive (%)	Intermediate (%)	Resistant (%)
*Viridans group Streptococcus sp.*	1 (100.0)	0 (0.0)	0 (0. 0)

Frequency calculated in relation to the participants who performed the antibiogram

**Table 12:** Antibiotic sensitivity to ciprofloxacin

	Sensitive (%)	Intermediate (%)	Resistant (%)
*Acinetobacter baumannii-calcoacetius complex*	0 (0.0)	2 (66.7)	1 (33.3)
*A. lwoffii*	0 (0.0)	1 (100.0)	0 (0.0)
*Corynebacterium sp*.	1 (100.0)	0 (0.0)	0 (0.0)
*Escherichia coli*	1 (50.0)	0 (0.0)	1 (50.0)
*Klebsiella oxytoca*	3 (100.0)	0 (0.0)	0 (0.0)
*K. pneumoniae*	2 (100.0)	0 (0.0)	0 (0.0)
*Morganella morganii*	1 (100.0)	0 (0.0)	0 (0.0)
*Serratia marcescens*	1 (100.0)	0 (0.0)	0 (0.0)
*Staphylococcus aureus*	0 (0.0)	11 (73.3)	4 (26.7)
*S. capitis*	0 (0.0)	1 (100.0)	0 (0.0)
*S. epidermidis*	0 (0.0)	12 (38.7)	19 (61.3)
*S. haemolyticus*	0 (0.0)	5 (41.7)	7 (58.3)
*S. hominis*	0 (0.0)	1 (100.0)	0 (0.0)
*S. saprophyticus*	0 (0.0)	1 (100.0)	0 (0.0)
*Staphylococcus sp. (coagulase negative)*	0 (0.0)	22 (43.1)	29 (56.9)

Frequency calculated in relation to the participants who performed the antibiogram

**Table 13:** Microorganism sensitivity to clarithromycin

	Sensitive (%)	Intermediate (%)	Resistant (%)
*Corynebacterium sp.*	1 (100.0)	0 (0.0)	0 (0.0)
*Klebsiella pneumoniae*	0 (0.0)	0 (0.0)	1 (100.0)
*Staphylococcus aureus*	1 (6.7)	0 (0.0)	14 (93.3)
*S. capitis*	0 (0.0)	0 (0.0)	1 (100.0)
*S. epidermidis*	7 (22.6)	0 (0.0)	24 (77.4)
*S. haemolyticus*	5 (41.7)	0 (0.0)	7 (58.3)
*S. hominis*	0 (0.0)	0 (0.0)	1 (100.0)
*S. saprophyticus*	1 (100.0)	0 (0.0)	0 (0.0)
*Staphylococcus sp. (coagulase negativo)*	20 (39.2)	0 (0.0)	31 (60.8)

Frequency calculated in relation to the participants who performed the antibiogram

**Table 14:** Microorganism sensitivity to clindamycin

	Sensitive (%)	Intermediate (%)	Resistant (%)
*Corynebacterium sp.*	1 (100.0)	0 (0.0)	0 (0.0)
*Staphylococcus aureus*	4 (26.7)	0 (0.0)	11 (73.3)
*S.capitis*	1 (100.0)	0 (0.0)	0 (0.0)
*S. epidermidis*	19 (61.3)	0 (0.0)	12 (38.7)
*S. haemolyticus*	8 (66.7)	0 (0.0)	4 (33.3)
*S. hominis*	0 (0.0)	0 (0.0)	1 (100.0)
*S. saprophyticus*	1 (100.0)	0 (0.0)	0 (0.0)
*Staphylococcus sp. (coagulase negative)*	39 (76.5)	0 (0.0)	12 (23.5)
*Viridans group Streptococcus sp.*	3 (100.0)	0 (0.0)	0 (0.0)

Frequency calculated in relation to the participants who performed the antibiogram

**Table 15:** Microorganism sensitivity to doxycycline

	Sensitive (%)	Intermediate (%)	Resistant (%)
*Corynebacterium sp.*	1 (100.0)	0 (0.0)	0 (0.0)
*Staphylococcus aureus*	11 (100.0)	0 (0.0)	0 (0.0)
*S. capitis*	1 (100.0)	0 (0.0)	0 (0.0)
*S. epidermidis*	24 (100.0)	0 (0.0)	0 (0.0)
*S. haemolyticus*	11 (100.0)	0 (0.0)	0 (0.0)
*S. hominis*	1 (100.0)	0 (0.0)	0 (0.0)
*S. saprophyticus*	1 (100.0)	0 (0.0)	0 (0.0)
*Staphylococcus sp. (coagulase negative)*	46 (100.0)	0 (0.0)	0 (0.0)

Frequency calculated in relation to the participants who performed the antibiogram

**Table 16:** Microorganism sensitivity to ertapenem

	Sensitive (%)	Intermediate (%)	Resistant (%)
*Escherichia coli*	2 (100.0)	0 (0.0)	0 (0.0)
*Klebsiella oxytoca*	3 (100.0)	0 (0.0)	0 (0.0)
*K. pneumoniae*	2 (100.0)	0 (0.0)	0 (0.0)
*Morganella morganii*	1 (100.0)	0 (0.0)	0 (0.0)
*Serratia marcescens*	1 (100.0)	0 (0.0)	0 (0.0)
*Viridans group Streptococcus sp.*	3 (100.0)	0 (0.0)	0 (0.0)

Frequency calculated in relation to the participants who performed the antibiogram

**Table 17:** Microorganism sensitivity to imipenem

	Sensitive (%)	Intermediate (%)	Resistant (%)
*Acinetobacter baumannii-caalcoacetius complex*	3 (100.0)	0 (0.0)	0 (0.0)
*A. lwoffii*	1 (100.0) (100.0)	0 (0.0)	0 (0.0)
*Viridans group Streptococcus sp*	2 (100.0)		0 (0.0)

Frequency calculated in relation to the participants who performed the antibiogram

**Table 18:** Microorganism sensitivity to erythromycin

	Sensitive (%)	Intermediate (%)	Resistant (%)
*Corynebacterium sp.*	1 (100.0)	0 (0.0)	0 (0.0)
*Klebsiella pneumoniae*	0 (0.0)	0 (0.0)	1 (100.0)
*Staphylococcus aureus*	1 (6.7)	0 (0.0)	14 (93.3)
*S. capitis*	0 (0.0)	0 (0.0)	1 (100.0)
*S. epidermidis*	9 (29.0)	0 (0.0)	22 (71.0)
*S. haemolyticus*	5 (41.7)	0 (0.0)	7 (58.3)
*S. hominis*	0 (0.0)	0 (0.0)	1 (100.0)
*S. saprophyticus*	1 (100.0)	0 (0.0)	0 (0.0)
*Staphylococcus sp. (coagulase negative)*	19 (37.3)	0 (0.0)	32 (62.7)
*Viridans group Streptococcus sp.*	0 (0.0)	0 (0.0)	1 (100.0)

Frequency calculated in relation to the participants who performed the antibiogram

**Table 19:** Microorganism sensitivity to streptomycin

	Sensitive (%)	Intermediate (%)	Resistant (%)
*Enterococcus faecalis*	1 (50.0)	0 (0.0)	1 (50.0)
*Staphylococcus aureus*	1 (100.0)	0 (0.0)	0 (0.0)

Frequency calculated in relation to the participants who performed the antibiogram

**Table 20:** Microorganism sensitivity to gentamicin

	Sensitive (%)	Intermediate (%)	Resistant (%)
*Acinetobacter baumannii-calcoacetius complex*	3 (100.0)	0 (0.0)	0 (0.0)
*A. lwoffii*	1 (100.0)	0 (0.0)	0 (0.0)
*Corynebacterium sp.*	1 (100.0)	0 (0.0)	0 (0.0)
*Enterococcus faecalis*	2 (100.0)	0 (0.0)	0 (0.0)
*Escherichia coli*	2 (100.0)	0 (0.0)	0 (0.0)
*Klebsiella oxytoca*	3 (100.0)	0 (0.0)	0 (0.0)
*K. pneumoniae*	2 (100.0)	0 (0.0)	0 (0.0)
*Morganella morganii*	1 (100.0)	0 (0.0)	0 (0.0)
*Serratia marcescens*	1 (100.0)	0 (0.0)	0 (0.0)
*Staphylococcus aureus*	12 (80.0)	1 (6.7)	2 (13.3)
*S. capitis*	1 (100.0)	0 (0.0)	0 (0.0)
*S. epidermidis*	21 (72.4)	0 (0.0)	8 (27.6)
*S. haemolyticus*	10 (83.3)	0 (0.0)	2 (16.7)
*S. hominis*	1 (100.0)	0 (0.0)	0 (0.0)
*S. saprophyticus*	1 (100.0)	0 (0.0)	0 (0.0)
*Staphylococcus sp. (coagulase negative*)	39 (76.5)	0 (0.0)	12 (23.5)

Frequency calculated in relation to the participants who performed the antibiogram

**Table 21:** Microorganism sensitivity to levofloxacin

	Sensitive (%)	Intermediate (%)	Resistant (%)
*Acinetobacter baumannii-calcoacetius complex*	2 (66.7)	0 (0.0)	1 (33.3)
*A. lwoffii*	1 (100.0)	0 (0.0)	0 (0.0)
*Corynebacterium sp.*	1 (100.0)	0 (0.0)	0 (0.0)
*Staphylococcus aureus*	0 (0.0)	11 (73.3)	4 (26.7)
*S. capitis*	0 (0.0)	1 (100.0)	0 (0.0)
*S. epidermidis*	0 (0.0)	12 (38.7)	19 (61.3)
*S. haemolyticus*	1 (8.3)	2 (16.7)	9 (75.0)
*S. hominis*	0 (0.0)	1 (100.0)	0 (0.0)
S. saprophyticus	0 (0.0)	1 (100.0)	0 (0.0)
*Staphylococcus sp. (coagulase negative)*	0 (0.0)	21 (42.9)	28 (57.1)

Frequency calculated in relation to the participants who performed the antibiogram

**Table 22:** Microorganism sensitivity to linezolid

	Sensitive (%)	Intermediate (%)	Resistant (%)
*Corynebacterium sp.*	1 (100.0)	0 (0.0)	0 (0.0)
*Enterococcus faecalis*	2 (100.0)	0 (0.0)	0 (0.0)
*Staphylococcus aureus*	15 (100.0)	0 (0.0)	0 (0.0)
*S. capitis*	1 (100.0)	0 (0.0)	0 (0.0)
*S. epidermidis*	31 (100.0)	0 (0.0)	0 (0.0)
*S. haemolyticus*	12 (100.0)	0 (0.0)	0 (0.0)
*S. saprophyticus*	1 (100.0)	0 (0.0)	0 (0.0)
*Staphylococcus sp. (coagulase negative)*	49 (100.0)	0 (0.0)	0 (0.0)

Frequency calculated in relation to the participants who performed the antibiogram

**Table 23:** Microorganism sensitivity to oxacillin

	Sensitive (%)	Intermediate (%)	Resistant (%)
*Corynebacterium sp.*	1 (100.0)	0 (0.0)	0 (0.0)
*Klebsiella pneumoniae*	1 (100.0)	0 (0.0)	0 (0.0)
*Staphylococcus aureus*	13 (86.7)	0 (0.0)	2 (13.3)
*S. capitis*	1 (100.0)	0 (0.0)	0 (0.0)
*S. epidermidis*	13 (41.9)	0 (0.0)	18 (58.1)
*S. haemolyticus*	4 (33.3)	0 (0.0)	8 (66.7)
*S. hominis*	0 (0.0)	0 (0.0)	1 (100.0)
*S. saprophyticus*	1 (100.0)	0 (0.0)	0 (0.0)
*Staphylococcus sp. (coagulase negative)*	29 (58.0)	0 (0.0)	21 (42.0)
*Viridans group Streptococcus sp.*	1 (100.0)	0 (0.0)	0 (0.0)

Frequency calculated in relation to the participants who performed the antibiogram

**Table 24:** Microorganism sensitivity to penicillin

	Sensitive (%)	Intermediate (%)	Resistant (%)
*Corynebacterium sp.*	1 (100.0)	0 (0.0)	0 (0.0)
*Klebsiella pneumoniae*	0 (0.0)	0 (0.0)	1 (100.0)
*Staphylococcus aureus*	0 (0.0)	0 (0.0)	15(100.0)
*S. capitis*	0 (0.0)	0 (0.0)	1 (100.0)
*S.epidermidis*	2 (6.5)	0 (0.0)	29 (93.5)
*S.haemolyticus*	0 (0.0)	0 (0.0)	12 (100.0)
*S. hominis*	0 (0.0)	0 (0.0)	1 (100.0)
*S. saprophyticus*	0 (0.0)	0 (0.0)	1 (100.0)
*Staphylococcus sp. (coagulase negative)*	4 (8.0)	0 (0.0)	46 (92.0)
*Viridans group Streptococcus sp.*	2 (66.7)	0 (0.0)	1 (33.3)

Frequency calculated in relation to the participants who performed the antibiogram

**Table 25:** Microorganism sensitivity to rifampicin

	Sensitive (%)	Intermediate (%)	Resistant (%)
*Corynebacterium sp.*	1 (100.0)	0 (0.0)	0 (0.0)
*Staphylococcus aureus*	15 (100.0)	0 (0.0)	0 (0.0)
*S. capitis*	1 (100.0)	0 (0.0)	0 (0.0)
*S. epidermidis*	31 (100.0)	0 (0.0)	0 (0.0)
*S. haemolyticus*	12 (100.0)	0 (0.0)	0 (0.0)
*S. hominis*	1 (100.0)	0 (0.0)	0 (0.0)
*S. saprophyticus*	1 (100.0)	0 (0.0)	0 (0.0)
*Staphylococcus sp. (coagulase negative)*	48 (96.0)	0 (0.0)	2 (4.0)

Frequency calculated in relation to the participants who performed the antibiogram

**Table 26:** Microorganism sensitivity to sulfamethoxazole-trimetroprim

	Sensitive (%)	Intermediate (%)	Resistant (%)
*Acinetobacter baumannii-calcoacetius complex*	0 (0.0)	0 (0.0)	3 (100.0)
*A. lwoffii*	1 (100.0)	0 (0.0)	0 (0.0)
*Corynebacterium sp.*	1 (100.0)	0 (0.0)	0 (0.0)
*Escherichia coli*	1 (50.0)	0 (0.0)	1 (50.0)
*Klebsiella oxytoca*	3 (100.0)	0 (0.0)	0 (0.0)
*K. pneumoniae*	2 (100.0)	0 (0.0)	0 (0.0)
*Morganella morganii*	1 (100.0)	0 (0.0)	0 (0.0)
*Serratia marcescens*	1 (100.0)	0 (0.0)	0 (0.0)
*Staphylococcus aureus*	15 (100.0)	0 (0.0)	0 (0.0)
*S. capitis*	1 (100.0)	0 (0.0)	0 (0.0)
*S. epidermidis*	29 (93.5)	0 (0.0)	2 (6.5)
*S. haemolyticus*	9 (75.0)	0 (0.0)	3 (25.0)
*S. hominis*	1 (100.0)	0 (0.0)	0 (0.0)
*S. saprophyticus*	1 (100.0)	0 (0.0)	0 (0.0)
*Streptococcus sp*. *(coagulase negative)*	43 (86.0)	0 (0.0)	7 (14.0)
*Viridans group Streptococcus sp.*	1 (100.0)	0 (0.0)	0 (0.0)

Frequency calculated in relation to the participants who performed the antibiogram

**Table 27:** Microorganism sensitivity to tetracycline

	Sensitive (%)	Intermediate (%)	Resistant (%)
*Corynebacterium sp.*	1 (100.0)	0 (0.0)	0 (0.0)
*Staphylococcus aureus*	11 (73.3)	0 (0.0)	4 (26.7)
*S. capitis*	1 (100.0)	0 (0.0)	0 (0.0)
*S. epidermidis*	26 (83.9)	0 (0.0)	5 (16.1)
*S. haemolyticus*	11 (91.7)	0 (0.0)	1 (8.3)
*S.hominis*	1 (100.0)	0 (0.0)	0 (0.0)
*S. saprophyticus*	1 (100.0)	0 (0.0)	0 (0.0)
*Staphylococcus sp. (coagulase negative)*	45 (90.0)	0 (0.0)	5 (10.0)

Frequency calculated in relation to the participants who performed the antibiogram

**Table 28:** Microorganism sensitivity to vancomycin

	Sensitive (%)	Intermediate (%)	Resistant (%)
*Corynebacterium sp.*	1 (100.0)	0 (0.0)	0 (0.0)
*Enterococcus faecalis*	1 (100.0)	0 (0.0)	0 (0.0)
*Staphylococcus aureus*	15 (100.0)	0 (0.0)	0 (0.0)
*S. capitis*	1 (100.0)	0 (0.0)	0 (0.0)
*S. epidermidis*	31 (100.0)	0 (0.0)	0 (0.0)
*S.s haemolyticus*	12 (100.0)	0 (0.0)	0 (0.0)
*S.s hominis*	1 (100.0)	0 (0.0)	0 (0.0)
*S. saprophyticus*	1 (100.0)	0 (0.0)	0 (0.0)
*Staphylococcus sp. (coagulase negative)*	50 (100.0)	0 (0.0)	0 (0.0)
*Viridans group Streptococcus sp.*	2 (100.0)	0 (0.0)	0 (0.0)

Frequency calculated in relation to the participants who performed the antibiogram

**Table 29:** Microorganism sensitivity to tobramycin

	Sensitive (%)	Intermediate (%)	Resistant (%)
*Corynebacterium sp.*	1 (100.0)	0 (0.0)	0 (0.0)
*Escherichia coli*	1 (100.0)	0 (0.0)	0 (0.0)
*Klebsiella oxytoca*	2 (100.0)	0 (0.0)	0 (0.0)
*Serratia marcescens*	1 (100.0)	0 (0.0)	0 (0.0)
*Staphylococcus aureus*	12 (100.0)	0 (0.0)	0 (0.0)
*S. epidermidis*	14 (70.0)	0 (0.0)	6 (30.0)
*S. haemolyticus*	5 (100.0)	0 (0.0)	0 (0.0)
*S. saprophyticus*	1 (100.0)	0 (0.0)	0 (0.0)
*Staphylococcus sp. (coagulase negative)*	27 (93.1)	0 (0.0)	2 (6.9)
*Viridans group Streptococcus sp.*	1 (100.0)	0 (0.0)	0 (0.0)

Frequency calculated in relation to the participants who performed the antibiogram

After positive cultures were identified, antibiograms were performed to determine the sensitivity to amikacin, amoxicillin, ampicillin, azithromycin, cefepime, cefotaxime, ceftriaxone, cefuroxime, ciprofloxacin, clarithromycin, clindamycin, doxycycline, ertapenem, imipenem, erythromycin, streptomycin, gentamicin, levofloxacin, linezolid, oxacillin, penicillin, rifampicin, trimethoprim-sulfamethoxazole, tetracycline, vancomycin, and tobramycin.

All bacteria were sensitive to amikacin (Table 4); the microorganisms resistance to azithromycin were as follows: 93.3% of the samples of *S. aureus*, 100% of *S. capitis*, 71% of *S. epidermidis*, 58.3% of *S. haemolyticus*, 100% of *S. hominis*, and 60.8% of *Staphylococcus sp.* (coagulase negative) (Table 7); all *Escherichia coli* samples were resistant to cefepime, cefotaxime, and ceftriaxone, and there was 50% of *E. coli* was resistant (Tables 8, 9, 10); the microorganisms resistant to ciprofloxacin were *Acinetobacter baumannii-calcoacetius complex* (33%), *E. coli* (50%), *S. aureus* (26.7%), *S. epidermidis* (61.3%), *S. haemolyticus* (58.3%) and *Staphylococcus* sp. (coagulase negative, 56.9%), (Table 12); all microorganisms had 100% sensitivity to doxycycline, ertapenem, and imipenem (Tables 15, 16, 17); the microorganisms resistant to levofloxacin were *A. baumannii-calcoacetius complex* (33.3%), *S. aureus* (26.7%), *S. epidermidis* (61.3%), *S. haemolyticus* (75%), and *Staphylococcus sp.* (coagulase negative, 57.1%), (Table 21); 100% of bacteria were sensitive to linezolid (22); the microorganisms resistant to oxacillin were *S. aureus* (13.3%), *S. epidermidis* (58.1%), *S. haemolyticus* (66.7%), *S. hominis* (100%) and *Staphylococcus sp.* (coagulase negative, 42%) (Table 23); 107 of 116 samples were resistant to penicillin: *K. pneumoniae* (100%), *S. aureus* (100%), *S. capitis* (100%), *S. epidermidis* (93.5%), *S. haemolyticus* (100%), *S. hominis* (100%), *S. saprophyticus* (100%), *Staphylococcus sp.* (coagulase negative, 92%), and viridans group *Streptococcus sp.* (33.3%) (Table 24).

Regarding the results of antibiograms performed for rifampicin, all samples were sensitive, with the exception of two samples of *Staphylococcus sp.* (coagulase negative, 4%) (Table 25); the microorganisms resistant to sulfamethoxazole-trimetroprim were *A. baumannii-calcoacetius complex* (100%), *E. coli* (50%), *S. epidermidis* (6.5%), *S. haemolyticus* (25%), and *Staphylococcus sp.* (coagulase negative, 14%) (Table 26).

The microorganisms resistant to tetracycline were *S. aureus* (26.7%), *S. epidermidis* (16.1%), *S. haemolyticus* (8.3%). and *Staphylococcus sp.* (coagulase negative, 10%) (Table 27).

All 115 bacterial samples taken to perform the vancomycin antibiogram demonstrated a sensitive profile (Table 28); those resistant to tobramycin were six samples of *S. epidermidis* (30%) and two samples of *Staphylococcus sp.* (coagulase negative, 6.9%); the remaining 65 samples taken for the antibiogram were sensitive to tobramycin (Table 29).

## Discussion

Technological advances in retinal and vitreous subspecialties have increased the diagnosis of pathologies and the indications for intravitreal injections, contributing to a global rise in the treatment of chronic retinal conditions requiring long-term therapy [27]. While prophylactic topical antibiotic therapy after intravitreal injections may seem beneficial for reducing endophthalmitis risk, the Diabetic Retinopathy Clinical Research Network reported an incidence of 1 in 3,333 injections without topical antibiotics, compared to six cases in 4,694 injections with antibiotic eye drops [28]. Similarly, a 2011 study in *Retina* showed comparable incidence rates: five cases in 2,480 patients without eye drops and five in 2287 with them [29]. Furthermore, patients using antibiotic drops had a higher risk of endophthalmitis with worse clinical outcomes due to the resistance profile associated with chronic antibiotic use [30–32]. Our study found 54 cultures (36.48%) positive for coagulase-negative *Staphylococcus* and 33 (22.29%) for *S. epidermidis*, with 100% sensitivity to vancomycin. Coagulase-negative *Staphylococcus* also showed 76.5% sensitivity to gentamicin, similar to findings in previous studies [34]. Resistance profiles indicated that 92% of coagulase-negative *Staphylococcus* were resistant to penicillin, closely aligning with the 95% resistance reported by Keshav and Basu et al. [34].

Kim and Toma reported a significant risk of selecting resistant strains of coagulase-negative *Staphylococcus* with antibiotic prophylaxis using macrolides or fluoroquinolones compared to treatment-naïve eyes (94% vs. 50%, P = 0.009) [33]. At the Wills Eye Institute in Philadelphia, Hsu and colleagues studied 25 conjunctival samples positive for *Staphylococcus* from patients receiving only povidone-iodine prophylaxis and found no resistance to macrolides or fluoroquinolones [19].

Some cases of endophthalmitis can be treated with vitrectomy or intravitreal antibiotic injection [13], typically using vancomycin (a glycopeptide) and ceftazidime (a third-generation cephalosporin). In our study, all 115 bacterial samples were sensitive to vancomycin, and third-generation cephalosporins showed high sensitivity, with only one resistant sample among 11 tested. This supports established protocols for treating endophthalmitis with vancomycin and ceftazidime. However, second-generation quinolones like ciprofloxacin and ofloxacin exhibited higher resistance rates in common conjunctival bacteria: *S. aureus* (26.7%), *S. epidermidis* (61.3%), and coagulase-negative *Staphylococcus* (56.9%). This resistance pattern with quinolones contrasts with the sensitivity observed for vancomycin and ceftriaxone, weakening the hypothesis of cross-resistance and reducing the risk of multidrug-resistant endophthalmitis despite antibiotic prophylaxis.

While the relationship between the use of PVPI and the selection of more virulent strains has been suggested, this theory has not been thoroughly explored in ophthalmology [31]. Other studies indicate that cross-resistance to antibiotic therapy may develop through the use of various biocidal solutions, including chlorhexidine, quaternary ammonium compounds, and topical PVPI [35–40]. However, only one study involving cultures from patients with peritoneal dialysis, who used this biocide daily, reported bacterial resistance linked to topical PVPI [41]. No clinical ophthalmologic study has demonstrated an association between topical PVPI and increased antibiotic resistance of conjunctival microbiota without the concurrent use of topical ocular antibiotics [19].

A study conducted in India found that exposure to subinhibitory concentrations of prophylactic antibiotics may lead to the emergence of new strains with increased resistance and virulence [42]. In that study, no positive cultures were observed in the group without topical antibiotics, while 73% of coagulase-negative *Staphylococcus* samples from the antibiotic group showed fluoroquinolone resistance. In contrast, our study reported 16 of 148 cultures as negative (10.81%), with 29 of 51 (56.9%) coagulase-negative *Staphylococcus* samples resistant to fluoroquinolones, a lower resistance rate than in the Indian study [42, 43].

The limitations of our current study include the small number of antibiograms performed for cephalosporins. It would also be valuable to test ceftazidime, an antibiotic used to treat endophthalmitis, which was unavailable through the laboratory at the Faculty of Medicine of ABC.

## Conclusions

Povidone-iodine (PVPI) is the gold standard for antisepsis during intravitreal injections, with literature showing no additional benefits from topical antibiotic prophylaxis. Our study found fewer microorganisms resistant to quinolones compared to other populations, but with a similar sensitivity pattern to vancomycin and resistance to penicillin. The higher resistance to quinolones in normal conjunctival microbiota suggests microbial selection, a widely discussed phenomenon. This study emphasizes the importance of patient safety and evidence-based medicine, especially as several countries, including Brazil, prescribe antibiotic eye drops post-injection, potentially leading to the selection of more virulent and quinolone-resistant strains. We found no evidence supporting cross-selection; our samples showed high sensitivity to vancomycin and ceftriaxone, critical antibiotics for treating endophthalmitis. The significance of this study lies in promoting awareness of microbial resistance, clinical protocols following intravitreal injections, community microbiota, and patient safety, ultimately enhancing understanding of antibiotic therapy’s immediate and long-term consequences.

## Data Availability

No datasets were generated or analysed during the current study.
